# Correction: Photoswitchable dynasore analogs to control endocytosis with light

**DOI:** 10.1039/d0sc90189j

**Published:** 2020-09-01

**Authors:** Núria Camarero, Ana Trapero, Ariadna Pérez-Jiménez, Eric Macia, Alexandre Gomila-Juaneda, Andrés Martín-Quirós, Laura Nevola, Artur Llobet, Amadeu Llebaria, Jordi Hernando, Ernest Giralt, Pau Gorostiza

**Affiliations:** a Institute for Bioengineering of Catalonia (IBEC) , The Barcelona Institute of Science and Technology (BIST) , Spain . Email: pau@icrea.cat; b Institute for Advanced Chemistry of Catalonia (IQAC-CSIC) , Spain; c Institut de Pharmacologie Moléculaire et Cellulaire (IPMC) , Université Nice Sophia Antipolis , France; d Institute for Research in Biomedicine (IRB Barcelona) , Spain; e Bellvitge Biomedical Research Institute (IDIBELL) , L’Hospitalet de Llobregat , Barcelona , Spain; f Department of Pathology and Experimental Therapy , Faculty of Medicine and Health Science , Institute of Neurosciences , University of Barcelona , 08907 L’Hospitalet de Llobregat , Barcelona , Spain; g Departament de Química , Universitat Autònoma de Barcelona (UAB) , Spain; h Universitat de Barcelona (UB) , Spain; i CIBER-BBN , Spain; j ICREA , Spain

## Abstract

Correction for ‘Photoswitchable dynasore analogs to control endocytosis with light’ by Núria Camarero *et al.*, *Chem. Sci.*, 2020, **11**, 8981–8988, DOI: ; 10.1039/d0sc03820b.



## 

The authors regret an error in the affiliation of one of the authors, Artur Llobet, in the original manuscript. The corrected list of authors and affiliations for this paper is as shown in this Correction article.

The Royal Society of Chemistry apologises for these errors and any consequent inconvenience to authors and readers.

